# Thyroid and renal tumors in patients treated with long-term lithium: case series from a lithium clinic, review of the literature and international pharmacovigilance reports

**DOI:** 10.1186/s40345-018-0125-9

**Published:** 2018-08-06

**Authors:** Luca Ambrosiani, Claudia Pisanu, Arianna Deidda, Caterina Chillotti, Maria Erminia Stochino, Alberto Bocchetta

**Affiliations:** 1 Section of Neuroscience and Clinical Pharmacology, Department of Biomedical Sciences, University of Cagliari, S.S. 554, km 4,500, Monserrato, Italy; 2 Sardinian Regional Centre of Pharmacovigilance, Azienda Ospedaliero-Universitaria, Cagliari, Italy; 3 Unit of Clinical Pharmacology, “San Giovanni di Dio Hospital”, Azienda Ospedaliero-Universitaria, Cagliari, Italy

**Keywords:** Lithium, Thyroid cancer, Renal cancer, VigiAccess, EudraVigilance

## Abstract

**Background:**

Cancer had never been considered as a relevant problem in patients treated with lithium until 2015, when a document published by the European Medicine Agency concluded that long-term use of lithium might induce renal tumors. A few months later, we observed the case of a woman treated with lithium for 18 years who was diagnosed with both thyroid and renal tumors.

**Methods:**

This study aimed to investigate the correlation between lithium treatment and thyroid or renal tumors. We analyzed clinical records in our lithium clinic database, causes of death of patients who had been visited at least once at the lithium clinic, reports of lithium adverse reactions in the European and WHO pharmacovigilance databases, and published cases of thyroid and renal tumors in long-term lithium-treated patients.

**Results:**

Of the 1871 lithium patients who had been visited at least once between 1980 and 2013, eight had been diagnosed with thyroid papillary carcinoma and two with clear-cell renal-cell carcinoma. No cases of thyroid cancer and only one case of renal tumor were the cause of death according to the 375 available death certificates. VigiAccess database contained a total of 29 and 14 cases of renal and thyroid tumors, respectively. EudraVigilance database contained 21 cases of renal and 8 of thyroid neoplasms. Literature search yielded 6 published cases of thyroid papillary carcinoma and 25 cases of various renal tumors. However, two population-based studies did not find any increased risks of cancer in patients exposed to lithium, whereas two nationwide studies did not find any excess of renal tumors.

**Conclusion:**

So far it has not been possible epidemiologically to confirm an increased risk of thyroid or renal cancers associated with lithium. Such a conclusion is supported by the findings of low rates and mortalities of thyroid or renal cancers from the present lithium clinic data.

## Background

Lithium has been essential in the maintenance therapy of patients with recurrent mood disorders since the discovery of its effects in mania in 1949 and in the prophylaxis of manic depression thereafter (for review see Bauer and Gitlin 2016). Lithium has also been shown to prevent suicide mortality, which is particularly high in patients with mood disorders (Lewitzka et al. 2015). Lithium is increasingly recommended as one of first line treatment or as the single preferred first-line drug in the long-term treatment of bipolar disorder (Nolen 2015).

Compared to common adverse events (for review see McKnight et al. 2012), cancer has long been considered a secondary problem in patients treated with lithium. However, the interest in the potential association between lithium therapy and tumors has recently been revived by a document issued by the European Medicine Agency (EMA) (2015), which adopted the following recommendation: “in light of the data available, the PRAC (Pharmacovigilance Risk Assessment Committee) has agreed that the evidence is sufficient to conclude that long-term use of lithium may induce microcysts, oncocytomas and collecting duct renal carcinomas. Therefore, the Marketing Authorisation Holders of lithium containing medicinal products should submit a variation within 2 months, to amend the product information. The document also recommends that routine pharmacovigilance should be performed in order to better characterize the risk (http://www.ema.europa.eu/docs/en_GB/document_library/PRAC_recommendation_on_signal/2015/01/WC500181043.pdf). The present study was prompted by a series of circumstances: (a) our group serves as the Sardinian Pharmacovigilance Center; (b) our group has been running a lithium clinic since the 1970s and is in possession of detailed clinical data regarding patients on maintenance treatment with lithium; (c) in 2015, a few months after the publication of the aforementioned EMA document, we observed the case of a woman treated with lithium for 18 years who was diagnosed with both thyroid and renal tumors.

We started this study aiming to investigate the possible correlation between lithium treatment and thyroid or renal tumors from different perspectives: (a) a retrospective analysis of the clinical records in the lithium clinic database; (b) an analysis of the causes of death of the patients who had been visited at least once at the lithium clinic between 1980 and 2013; (c) an analysis of the reports of lithium adverse reactions to the European and the WHO pharmacovigilance databases; and (d) a review of the literature on thyroid and renal tumors in patients treated with lithium.

## Methods

### Lithium clinic database

Clinical records of the patients in the database of the Unit of Clinical Pharmacology, Azienda Ospedaliero-Universitaria, Cagliari, were examined. Our Unit has been one of the reference centers for lithium monitoring in the Cagliari area since its introduction in the 1970s.

Lithium monitoring was in line with international guidelines. For the purpose of this study, we extracted the clinical data of patients who had been diagnosed with thyroid or renal tumors.

### Causes of death

In 2002, we started to analyze systematically mortality and causes of death of patients in our lithium clinic database (Bocchetta 2005; Bocchetta et al. 2007b). Life status was assessed through Population Registers from the last known town of residence. Population registers also constituted the source of death certificates up to 1984, whereas the Register of the Causes of Death of the Local Health Unit has been the source from 1985 onwards. We retrieved the information about life status for 1391 of 1411 (99%) patients in the database from 1980 to 2000. Death certificates were obtained for 191 of the 201 (95%) who had deceased up to 2002. Causes of death were reviewed and coded using the International Classification of Diseases-9th revision.

Mortality and causes of death were analyzed again in 2013. We retrieved the information about life status for 1826 of 1840 (99%) patients treated with lithium who had been visited at least once since 1980. Certificates of death were available for 375 cases.

### Pharmacovigilance databases

In January 2018 we searched VigiAccess, EudraVigilance. VigiAccess is a global database supervised by the Uppsala Monitoring Centre Vigibase, the World Health Organization (WHO) adverse drug reaction database (ADR) database. It provides free access to all individual case safety reports (ICSRs) spontaneously collected by over 110 national drug authorities (including Europe and US) since 1968, even though it does not allow analysis of the individual ADRs collected.

EudraVigilance is a European database managed by EMA, which is freely accessible and collects and processes suspected ADRs since December 2001. The databases were searched for the active ingredient “lithium”, and data were extracted according to the medical dictionary for regulatory activity (MedDRA). MedDRA terms are classified in system organ classes (SOC). Standardized MedDRA queries (SMQs) are tools developed to facilitate retrieval of MedDRA-coded data in pharmacovigilance.

Unfortunately, an official list of SMQs used for classifying thyroid and renal cancers is not available (https://www.meddra.org/standardised-meddra-queries). Therefore, any MedDRA term that could be related was searched under the SOC “neoplasms benign, malignant and unspecified”. General terms which did not relate to thyroid or kidney, such as “neoplasm”, “metastasis” or “adenocarcinoma” were excluded from the analysis. Cases of tumors of the renal pelvis were also excluded. Due to the overlap of the WHO and the European databases, a duplication of cases may have occurred.

### Review of the literature

A systematic review of the literature was conducted using PubMed and Scopus without date restrictions, to identify studies reporting cases of thyroid and renal tumors occurred in patients chronically treated with lithium. The search was conducted using the following search strategies with “English language” as a filter:

- “kidney tumor AND lithium”, “renal cancer AND lithium”, “renal carcinoma AND lithium”, “oncocytoma AND lithium”;
- combinations of the MeSH terms “kidney neoplasms”, “lithium/toxicity”, “cell proliferation/drug effects”, “lithium compounds/adverse effects”;
- “thyroid carcinoma AND lithium”, “thyroid tumor AND lithium”, “thyroid cancer AND lithium”;
- combinations of the MeSH terms “lithium compounds” AND “thyroid neoplasms”.

Bibliographic references of relevant articles were reviewed for additional studies.

Preclinical studies, as well as studies in which lithium was used as an adjuvant to ^131^I therapy of thyroid cancer were excluded, as were studies of non-malignant thyroid tumors (adenoma or goiter).

From each selected study we collected the following information: number of reported cases, pathological diagnosis, duration of exposure to lithium treatment. For renal tumors we also indicated, when available, presence of cysts and estimated glomerular filtration rate (eGFR).

## Results

### Case report

The following case was observed in 2015, a few months after the publication of the EMA document.

A 52-year-old woman had been treated with lithium carbonate, haloperidol and risperidone for schizoaffective disorder since 1997. The patient also received benzodiazepines. From 1998 to 2004, carbamazepine was added as a second mood stabilizer. Lithium doses varied throughout the years from 600 to 1050 mg/day, in order to keep serum concentration within the therapeutic range (0.6–0.8 mmol/L). No episodes of lithium intoxication occurred. Comorbidities included increased body mass index, repeated findings of abnormally high fasting plasma glucose, hypertension (treated with amlodipine, 5 mg/day). Over the last 10 years, her estimated glomerular filtration rate (eGFR) had gradually declined to 40 ml/min/1.73 m^2^, which falls into the category G3b [moderately to severely decreased chronic kidney disease (CKD)] according to KDIGO (kidney disease improving global outcomes) (KDIGO 2017). She developed a multinodular goiter that eventually caused dysphagia and dyspepsia. After an ultrasound scan, she underwent a medical workup in September 2015. Computer tomography scans showed abnormal thyroid images, multiple pulmonary metastasis, increased adrenal gland, a renal mass in the left kidney (maximum diameter, 38 mm), and multiple hyperdense nodules in the contralateral kidney (maximum diameter, 13 mm). She underwent thyroidectomy and the histological examination was consistent with the presence of two different tumors. A papillary thyroid carcinoma within adenomatous goiter tissue was identified within the left lobe, whereas an epithelial, sarcomatoid-like tumor was found in the right lobe. The latter was consistent with renal origin according to immunohistochemistry. A subsequent renal biopsy led to the diagnosis of oncocytoma, stage IV (M1). The patient died in May 2016 for respiratory failure due to pulmonary metastases.

We reported the case to the national pharmacovigilance agency in April 2016. The adverse reaction was codified as severe (life threatening). The causality assessment, evaluated by the Regional Centre of Pharmacovigilance according to the Naranjo algorithm (Naranjo et al. 1981), was considered “possible” based on the chronological criteria (18 years of lithium treatment), and the presence of objective evidence and previous conclusive reports. In fact, the ADR renal tumor has been included in the package leaflet since the 2015 EMA document.

### Lithium-clinic database

Of the 1871 patients treated with lithium who had been visited at least once between 1980 and 2013, 562 had been followed up with regular monitoring for at least 5 years (total = 7639 patient/years). The number of patients followed up for at least 10 years was 345 (total = 6212 patient/years).

Out of 19 (18 women, one man) who underwent thyroidectomy, 9 cases were diagnosed with thyroid cancer. The remaining were 9 cases of multinodular goiter and one case of adenoma. Characteristics of the patients with thyroid cancer are shown in Table 1. At the time of thyroidectomy, the median age of the patients was 53 years and their median duration of lithium therapy was 23 years.

**Table 1:** Characteristics of the 9 patients with thyroid cancer from our lithium clinic

Sex	Age at thyroidectomy	Years on lithium at thyroidectomy	Pathological diagnosis	Radio-iodine therapy
F	60	25	Papillary carcinoma, follicular variant, with focal invasion of the capsule	Yes
F	53	25	Papillary carcinoma	Yes
M	44	15	Papillary carcinoma, follicular variant	Yes
F	53	12	Papillary carcinoma	No
F	52	19	Papillary carcinoma^a^	No
F	54	26	Multifocal papillary carcinoma^b^	Yes
F	66	23	Papillary carcinoma	Yes
F	61	22	Papillary carcinoma	Yes
F	53	23	Papillary carcinoma	No

^a^Case report with concurrent renal oncocytoma described in this paper

^b^Case already reported in a follow-up study of a cohort of 150 patients (Bocchetta et al. 2007a)

With regard to renal tumors, only the following two cases were retrieved from our retrospective analysis of the lithium clinic database, besides the aforementioned case report.

A 58-year-old man had been diagnosed with clear-cell renal cancer and bilateral adrenal gland hyperplasia in 2003. He had been prescribed lithium since the age of 50 for Bipolar I Disorder. He also suffered from hypertension, dyslipidemia, hepatitis C virus infection with related chronic liver disease, and insulin-treated diabetes. He underwent nephrectomy and his eGFR decreased from 86 to 50 ml/min/1.73 m^2^. In 2005 he was also diagnosed with non-small-cell lung cancer and died in 2010 for cerebral metastases originated from the pulmonary neoplasm.

The second case regarded a man who had been treated with lithium since 1991 for Bipolar II Disorder. In November 2005, at age 55, during a medical work up including abdominal ultrasound examination, an abnormal image was found in the right kidney. After further investigation (including a CT scan) he underwent nephrectomy in September 2006. The histological diagnosis was clear-cell renal cancer. Thereafter, his eGFR decreased from 105 to 75 ml/min/1.73 m^2^ and lithium was replaced with valproate. The patient died in 2013 due to complications of his alcoholic cirrhosis which had been first diagnosed in 2006.

### Causes of death

Out of 375 death certificates regarding patients who had been visited at least once from 1980 to 2013, no cases of thyroid cancer and only one case of renal tumor were registered as the principal cause of death. The latter regarded a 68-year-old man who had been prescribed lithium 5 years earlier but had taken the therapy only for a few months.

The total number of patient/years calculated for the 1840 patients visited at least once from 1980 to 2013 amounted to 33,801, including 8263 patient/years on regular monitoring of lithium therapy. Causes of death were represented by other malignancies in 35 cases and by suicide in 64 cases (only one during regular maintenance with lithium).

### Pharmacovigilance databases

#### VigiAccess

A search on VigiAccess database performed in January 2018 retrieved a total of 22,280 lithium-related ADRs. Only 220 (1%) were classified under the System Organ Class (SOC) “neoplasms benign, malignant and unspecified”. We found a total of 29 and 14 individual cases of renal and thyroid tumors, respectively, codified with the MedDRA terms shown in Table 2.

**Table 2:** Number of reports of renal and thyroid neoplasms (classified by MedDRA terms) from the pharmacovigilance databases EudraVigilance and VigiAccess

	EudraVigilance	VigiAccess
Renal neoplasms, total cases	21	29
Renal-cell carcinoma	10	9
Oncocytoma	0	5
Renal oncocytoma	5	2
Renal cancer	4	5
Clear-cell renal-cell carcinoma	1	5
Renal neoplasm	1	2
Renal adenoma	0	1
Thyroid neoplasms, total cases	8	14
Thyroid cancer	2	9
Thyroid adenoma	5	0
Thyroid neoplasm	0	3
Medullary thyroid cancer	1	1
Benign neoplasm of thyroid gland	0	1

#### EudraVigilance

A search on EudraVigilance database performed in January 2018 retrieved a total of 9833 individual cases of ADRs related to lithium. Only 150 (1.53%) of them were classified as “neoplasms benign, malignant and unspecified”. Table 2 shows the MedDRA terms for those codified as renal (N = 21) or thyroid (N = 8) neoplasms. Demographic characteristics and outcome are shown in Table 3. All renal cases were reported by healthcare professionals, while non-healthcare professionals reported two of the 8 thyroid cases.

**Table 3:** Demographic characteristics and outcome in renal and thyroid neoplasm from the EudraVigilance database

	Renal neoplasms (N = 21)	Thyroid neoplasms (N = 8)
Sex		
Male	5	2
Female	12	6
Not specified	4	–
Age groups (years)		
18–64	7	7
65–85	10	1
Not specified	4	–
Outcome		
Not recovered	1	2
Recovered with sequelae	1	–
Recovered	6	2
Unknown	8	4
Not specified	5	–

### Review of the literature: thyroid tumors

Our search retrieved six articles identified as potentially relevant. Among the retrieved articles, one was excluded because it reported the case of a benign adenoma, one because it was not pertinent, while four articles were identified as meeting the inclusion criteria.

We only found six published cases of thyroid cancer in patients treated with lithium (Table 4). The first was observed in a 55-year-old woman who had been treated with lithium for 3.5 years. She presented with unilateral thyroid enlargement causing tracheal deviation. She underwent thyroidectomy and was diagnosed with a papillary-cell carcinoma. In particular, histological examination revealed a multinodular goiter with multiple areas of sclerosis with juxtaposed groups of malignant cells, some showing papillary structures. She was administered ^131^Iodine in an attempt to ablate the remnant thyroid tissue (Brownlie et al. 1980).

**Table 4:** Characteristics of thyroid tumors from the literature and from the present case series

Authors	N.	Pathological diagnosis	Years on lithium	Sex	Age	Notes
Brownlie et al. (1980)	1	Papillary-cell carcinoma	3.5	F	55	Multinodular goiter
McHenry et al. (1990)	3	Papillary carcinoma	3	F	48	Myxedema, multinodular goiter, radio-iodine therapy
		Papillary carcinoma	4	F	52	Myxedema, multinodular goiter
		Papillary carcinoma	6	F	78	Hypothyroidism, multinodular goiter, hyperparathyroidism
Aksoy et al. (2006)	1	Multifocal papillary carcinoma	4	M	45	Hypothyroidism, hashimoto thyroiditis
Bocchetta et al. (2007a), and present study	9	9 papillary carcinoma	12–26	8F 1M	44–66	Radio-iodine therapy in 6 cases

In a series of seven parathyroid or thyroid surgical cases in Canada, McHenry et al. (1990) reported three cases of thyroid carcinoma after long-term lithium therapy. All three cases were associated with multinodular goiter, hypothyroidism, and hyperparathyroidism (McHenry et al. 1990).

The fifth case was seen in a 45-year-old man who had been treated with lithium for 4 years who underwent thyroid workup for hypothyroidism. Fine needle aspiration revealed papillary carcinoma and the patient underwent thyroidectomy. Histopathological examination showed multifocal papillary carcinoma and Hashimoto thyroiditis (Aksoy et al. 2006).

The sixth published case was found in the context of a follow-up study of a cohort of 150 patients treated with lithium at our facility. It regarded a 54-year-old woman taking lithium since the age of 28. Histology revealed a multicentric papillary carcinoma. She was treated with ^131^Iodine after surgery (Bocchetta et al. 2007a).

### Review of the literature: renal tumors

Seventeen articles identified as potentially relevant were retrieved for evaluation of the full text. Among the retrieved articles, two were excluded because they considered renal tumors together with cancers of the renal pelvis or ureter, nine because they were not pertinent, while six articles were identified as meeting the inclusion criteria. Cases reported in these studies were excluded in case a histological diagnosis was missing. The six studies included in the review reported 25 cases of renal tumors occurred in patients chronically treated with lithium and meeting our inclusion criteria (Table 5). Among included cases, there was a high percentage of oncocytomas and collecting-ducts carcinomas, which are usually reported as uncommon or rare. In addition, the presence of cysts is very frequent, involving 72% of cases. The vast majority of cases (84%) presented an eGFR < 60 ml/min/1.73 m^2^ (CKD, G3 to G5). When the eGFR was not available, it was calculated from the plasma creatinine reported in the paper, using the Modification of Diet in Renal Disease (MDRD) Study Group equation (Levey et al. 1999).

**Table 5:** Case reports of histologically confirmed renal tumor associated with lithium treatment from the literature and from the present case series

Authors	Pathological diagnosis	Cysts	Years on lithium	Sex	Age	eGFR ml/min/1.73 m^2^ (CKD category)^a^
Markowitz et al. (2000) (2 cases)	Oncocytoma	+	20	M	50	13 (G5)
	Papillary renal-cell carcinoma	+	25	M	56	27 (G4)
Rookmaaker et al. (2012) (6 cases)	Collecting-ducts carcinoma	+	17	M	69	11 (G5)
	Collecting-ducts carcinoma	+	12	M	59	50 (G3a)
	Collecting-ducts carcinoma	+	15	M	61	48 (G3a)
	Oncocytoma	+	30	M	70	6 (G5)
	Oncocytoma	+	41	F	65	35 (G3b)
	Oncocytoma + collecting-ducts carcinoma	+	30	F	71	37 (3b)
Kjaersgaard et al. (2012)	Renal-cell carcinoma	+	28	M	71	Not available
Zardawi et al. (2013)	Renal-cell carcinoma	+	12	F	72	21 (G4)
Zaidan et al. (2014b) (14 cases)	Clear-cell renal-cell carcinoma	–	17	M	69	48 (G3a)
	Clear-cell renal-cell carcinoma	+	31	F	75	27 (G4)
	Clear-cell renal-cell carcinoma	–	18	F	70	24 (G4)
	Clear-cell renal-cell carcinoma + leiomyomatous stroma	–	41	F	71	55 (G3a)
	Oncocytomas + papillary adenomas	+	23	M	60	10 (G5)
	Oncocytoma	+	32	M	66	33 (G3b)
	Oncocytoma	+	16	M	68	52 (G3a)
	Oncocytoma	+	26	F	59	34 (G3b)
	Angiomyolipoma	–	21	F	65	24 (G4)
	Angiomyolipoma	–	>10	F	77	68 (G2)
	Papillary renal-cell carcinoma	+	15	M	56	52 (G3a)
	Papillary renal-cell carcinoma	–	35	M	54	61 (G2)
	Oncocytoma + chromophobe renal-cell carcinoma	+	10	F	66	5 (G5)
	Mixed epithelial and stromal tumor	+	3	F	46	71 (G2)
Jung (2016)	Renal-cell carcinoma	–	15	F	54	29 (G4)
This study (3 cases)	Clear-cell renal-cell carcinoma	–	8	M	56	86 (G2)
	Clear-cell renal-cell carcinoma	–	14	M	55	105 (G1)
	Oncocytoma^b^	–	18	F	52	40 (G3b)

^a^eGFR was calculated from reported serum creatinine concentrations using the Modification of Diet in Renal Disease Study Group equation (Levey et al. 1999). The following are the categories of chronic kidney disease (CKD) according to KDIGO (2017) based on eGFR: G1, “Normal or high” (≥ 90 ml/min/1.73 m^2^); G2, “Mildly decreased” (60–89 ml/min/1.73 m^2^); G3a, “Mildly to moderately decreased” (45–59 ml/min/1.73 m^2^); G3b, “Moderately to severely decreased” (30–44 ml/min/1.73 m^2^); G4, “Severely decreased” (15–29 ml/min/1.73 m^2^); G5, “Kidney failure” (< 15 ml/min/1.73 m^2^). It must be noted that KDIGO CKD stages are also based on albuminuria categories, which were not included in the present table

^b^Case with concurrent thyroid carcinoma described in this paper

Besides the case reports meeting the above-mentioned criteria, we retrieved two nationwide population-based studies that were prompted in order to estimate rates of renal tumors among individuals exposed to lithium.

Kessing et al. (2015) analyzed time-specific data from all individuals exposed to lithium (n = 24,272) or anticonvulsants (n = 386,255), all individuals with a diagnosis of bipolar disorder (n = 9651), and a randomly selected sample of 1500,000 from the Danish population. The study period was from 1995 to 2012. Analyses were adjusted for the number of prescriptions for lithium/anticonvulsants, antipsychotic agents, antidepressants, use of all other types of medication, sociodemographic variables, and a diagnosis of bipolar disorder. Continued treatment with lithium, but also with anticonvulsants, was not associated with increased rates of renal and upper urinary tract tumors.

Pottegård et al. (2016) identified all histologically verified upper urinary tract cancer cases in Denmark between 2000 and 2012 from the Danish Cancer Registry. A total of 6477 cases were matched by age and sex to 259,080 cancer-free controls. Data on lithium use from 1995 to 2012 were obtained from the Danish Prescription Registry. The study estimated the association between long-term use of lithium (≥ 5 years) and risk of upper urinary tract cancer using conditional logistic regression with adjustment for potential confounders. The number of renal cancers amounted to 11 cases among 5648 subjects exposed to lithium (1.9 per 1000 subjects) compared to 377 cases among 225,920 subjects not exposed to lithium (1.7 per 1000 subjects) resulting in non-significant odd ratios after adjustment for sociodemographic variables and comorbidities.

## Discussion

With this study, we sought to shed light on the issue of renal and thyroid cancer during long-term lithium therapy.

Of the 1871 lithium patients who had been visited at least once between 1980 and 2013, eight had been diagnosed with thyroid papillary carcinoma and two with clear-cell renal-cell carcinoma. No cases of thyroid cancer and only one case of renal tumor were the cause of death according to the 375 available death certificates.

Compared to other adverse events of lithium (McKnight et al. 2012), cancer has long been neglected until the publication of the 2015 EMA document. Indeed, very few clinical studies had investigated the association between cancer risk and lithium. There have been occasional reports of leukemia in patients treated with lithium (Lyskowski and Nasrallah 1981; Moreb and Hershko 1985; Volf and Crismon 1991), but a causal relationship has not yet been established. Cohen et al. (1998) studied 609 patients treated with lithium carbonate and 2396 controls. They found a non-significant lower risk to develop non-epithelial tumors among psychiatric patients treated with lithium as compared to controls. They also observed a significantly inverse trend of cancer occurrence with lithium dose. The risk of developing cancer among psychiatric patients was found to be significantly lower than in the general population and the study concluded that mental patients had a lower cancer prevalence and that lithium may exert a protective effect.

More recently, Huang et al. (2016) studied retrospectively a cohort of patients treated with lithium (N = 370) or anticonvulsants (N = 3250) for bipolar disorder in Taiwan. Lithium exposure was associated with significantly lower cancer risk compared with anticonvulsants exposure. They also observed a dose–response relationship for cancer risk reduction with lithium. For site-specific cancer, lithium users showed trends of decreased cancer risks, except for bone, skin, connective and other soft tissue as well as genitourinary cancer risk. However, the authors suggested to interpret these results with caution in light of the possibly limited statistical power due to the low number of specific cancer cases.

### Renal tumors

Apart from individual case reports (Table 5), the EMA document was principally based on two studies which were conducted in nephrology departments, one Dutch (Rookmaaker et al. 2012) and one French (Zaidan et al. 2014b).

Over a time period of 15 years, Rookmaaker et al. (2012) identified six patients with one or more solid renal tumors in their population of approximately 50 patients with chronic lithium-associated nephropathy. All patients were on lithium therapy for over 10 years and suffered from varying degrees of lithium nephropathy and renal diabetes insipidus. The renal tumors were incidental findings during workup for renal dysfunction or upon analysis of nonspecific abdominal complaints. The abdominal scans showed multiple uncomplicated renal cysts in all patients and a complicated cyst in the contralateral kidney in one patient. The tumors were all of collecting-duct origin and comprised both oncocytomas and collecting-duct carcinomas. The authors underscored that the latter differed from the “classical” collecting-duct-cell carcinomas, which are very rare with an incidence of less than 2 per 1000,000 person years (Storkel et al. 1997). They concluded that the 4 cases of collecting-duct-cell carcinomas in their population of approximately 50 patients with lithium nephropathy represent a much higher incidence. Other differences from the “classical” collecting-duct-cell carcinomas included histological appearance, multifocal presentation and non-aggressive clinical behavior. In fact, “classical” collecting-duct-cell carcinomas are aggressive tumors with 80% lymph node metastases at presentation and a median survival of only 22 months (Peyromaure et al. 2003).

Over a 16-year period, Zaidan et al. (2014b) found that 14 of 170 lithium-treated patients, followed at two nephrology departments in Paris and with available renal imaging, had various types of renal tumors (Table 5). The mean duration of lithium exposure at tumor diagnosis was above 20 years. Mean age at diagnosis was 64.4 ± 8.6 years. The percentage of renal tumors, particularly cancers and oncocytomas, was significantly higher in lithium treated patients compared with 340 gender-, age-, and eGFR-matched lithium free patients. Additionally, the Standardized Incidence Ratio of renal cancer was significantly higher in lithium treated patients compared with the French general population.

The EMA document has raised an intense debate, particularly in the context of the scientific community involved in the study of lithium therapy, because the epidemiological evidence leading to the recommendation was considered somewhat limited (Baldessarini and Tondo 2014; Licht et al. 2014; Zaidan et al. 2014a; Conell et al. 2015). For example, Licht et al. (2014) commented that, as all patients initiated on lithium (and controls) were not identified and followed over time, time at risk and cumulative events over time were not determined. Therefore, risks or incidence rate ratios as reported in the French study cannot be estimated. The observed difference in prevalence of tumors between the two patient samples may be explained by other factors than the effects of lithium, such as differential use of medications and differential referral patterns. Licht et al. concluded that no causal relationship between lithium and solid renal tumors can be inferred from the French study.

Zaidan et al. (2014a) replied that they had not estimated relative risks and incidence rate ratios between the two groups. Instead, they had calculated the number of expected cases of renal cancer in lithium-treated patients by gender and for each 5-year stratum using the French national estimates of renal cancer and showed increased standardized incidence ratios of renal cancers in lithium-treated patients compared to the general population. Moreover, they had reported an association and not a causal effect.

Baldessarini and Tondo (2014) commented that the French study did not report risks among lithium-treated patients without renal impairment. As the rates of severe impairment of renal function among lithium-treated patients may be as low as 0.5% (McKnight et al. 2012) and given an estimated risk of solid renal tumors of 8.24% of lithium-treated patients with significant renal impairment according to the French study, a tentative estimate of risk of kidney tumors among all patients exposed to lithium long-term would be approximately 0.041% (0.0824 × 0.005). This estimate suggests a risk of one case of solid renal tumor in approximately 2400 lithium-treated patients. However, as shown in Table 5, the patients referred to the French nephrology departments included cases with mildly decreased eGFR, so that Baldessarini and Tondo’s estimates are questionable. Moreover, McKnight et al.’s estimates are also questionable because they were based on prospective studies with a mean observation time of 1 year, whereas, in our survey, including patients followed for several decades, as many as half of the patients treated for longer than 20 years had an eGFR lower than 60 ml/min/1.73 m^2^ (Bocchetta et al. 2015).

In any case, Baldessarini and Tondo (2014) also mentioned that “based on consulting international colleagues who lead large mood disorder clinical programs, no reports of renal cancers have emerged in a collective experience involving thousands of psychiatric patients treated long-term with lithium (IGSLI centers, http://www.igsli.org; personal written communication, 28 March 2014; and clinical records of the Lucio Bini Mood Disorders Centers, Rome and Cagliari, March 2014).” The latter comment was one of the reasons that encouraged us to analyze retrospectively our lithium clinic database in order to overcome a potential recall bias. We thus identified the two cases of renal-cell cancer out of 1871 patients up to 2013. Curiously, the third case was observed in 2015, right after the publication of the EMA document.

Conell et al. (2015) warned about uncritical reactions including withdrawal of lithium in successfully treated patients after the adjustment within the lithium package leaflet was required by the European authorities concerning the increased risk for kidney tumors. We agree that any decision must weigh pros and cons.

The above-mentioned statements (Baldessarini and Tondo 2014; Licht et al. 2014; Zaidan et al. 2014a; Conell et al. 2015) were made prior to the two epidemiological studies clearly showing with overwhelming statistical power that long-term use of lithium is not associated with increased risk of renal tumors or cancer (Kessing et al. 2015; Pottegård et al. 2016).

### Thyroid cancer

As goiter was beyond the scope of this review, we found less literature on thyroid tumors in patients treated with lithium as compared to renal tumors. We retrieved only five case reports of thyroid cancer in addition to the one we had already reported in 2007 (Bocchetta et al. 2007a). The latter was found in the context of a follow-up study of a cohort of 150 patients at various stages of lithium treatment at our facility. We had detected nodules with ultrasonic scan in a large proportion of patients from that cohort (Licht et al. 2014). However, we observed only one case of malignancy during the 15-year follow-up (Bocchetta et al. 1996), concluding that it may be consistent with chance. In any case, we also acknowledged that, as further diagnostic evaluation of nodules (such as fine-needle aspiration biopsy) was not performed systematically, incidence might have been underestimated. Moreover, only three patients from that cohort had undergone thyroidectomy. Now, the present updated analysis of our entire cohort of lithium patients has revealed 16 additional cases of thyroidectomy, including the new 8 cases of thyroid cancer. All the nine cases observed (Table 1) were represented by papillary carcinoma and had occurred in middle-aged patients (mean 55 years) after an average of above 20 years of lithium exposure.

With our case series, the total number of reported cases amounts now to 14 (Table 4).

### Pharmacovigilance databases and epidemiological studies

We are aware that the number of ADR reports serves principally to generate or confirm an alarm, but its epidemiological value is limited. Reporting is subject to different influences, including the presence of previous literature reports. On the other hand, there may be overlapping across the different pharmacovigilance databases and with cases reported in the literature. We expect that the rate of reports of renal tumors is going to increase after the EMA document encouraged routine pharmacovigilance in order to better characterize the risk.

The analysis of data from the Pharmacovigilance systems has many limitations, including the underreporting and underestimation of the events and the impossibility to estimate the incidence of ADRs. Unfortunately, some ADRs were codified as “renal neoplasm” or “renal cancer”, with no histological characterization. The different terms used to classify the ADRs (e.g. oncocytoma, renal oncocytoma) further limit the analysis.

In any case, after the alarming finding of an increased risk of renal tumors among long-term lithium users, besides the routine pharmacovigilance reports, the two nationwide Danish population-based studies clearly showed with overwhelming statistical power that long-term use of lithium is not associated with increased risk of renal tumors or cancer (Kessing et al. 2015; Pottegård et al. 2016).

It appears that no epidemiological studies have addressed the issue of lithium-related thyroid cancer. We can just estimate an incidence of 9 cases over approximately 6200 patient/years in our cohort of 345 patients followed up for more than 10 years of lithium therapy.

It must be underscored that patients treated with lithium may receive a particular attention as regard to assessment of thyroid and renal functions, so that a surveillance bias cannot be ruled out in epidemiological studies. This issue was for example addressed by Licht et al. (2014): “lithium-treated patients are generally under careful observation for renal symptoms, even if not causally related to lithium, and non-lithium-treated patients may suffer from even serious renal problems that never lead to tertiary referral or renal imaging”.

### Potential mechanisms

Whatever the epidemiological relevance, some mechanisms potentially involved in lithium-related tumors are worth being discussed.

The cases of thyroid cancer reported so far in patients treated with lithium (Table 4) were invariably papillary carcinoma, which is the most common type also in the general population. Therefore, it cannot be concluded that lithium is a direct cause. However, given the known goitrogenic effect of lithium and the frequent finding of increased thyrotropin concentrations (for review see Bocchetta and Loviselli 2006), it is possible that lithium contributes to the propagation of incidental differentiated thyroid cancers. Moreover, it has been shown that lithium has a direct stimulatory effect on the growth of thyroid cells in culture (Tsuchiya et al. 1990).

The question of the potential role of lithium in renal tumors is somewhat different. In fact, beyond clear-cell renal-cell and papillary renal-cell cancer, other rare subtypes of renal cancers have been reported in patients treated with lithium (Table 5). Rookmaaker et al. (2012), who observed several cases of collecting-duct carcinoma, commented that the latter are derived from principal cells in the collecting duct, where lithium can reach very high concentrations. They hypothesized that a prolonged stimulation of principal cell proliferation by lithium, via its inhibition of the enzyme glycogen synthase kinase 3 beta and the increased availability of β-catenin in tubular cells (Kjaersgaard et al. 2012; Nielsen et al. 2008; Saadi-Kheddouci et al. 2001) can eventually induce adenomas and carcinomas.

On the other hand, the relatively high proportion of cases of the otherwise rare renal oncocytoma that have been reported in lithium patient is intriguing. Renal oncocytomas are derived from intercalated cells (Kuroda et al. 2003). Animal experiments have shown that lithium treatment not only induces the proliferation of principal cells, but also causes an increase in the number of intercalated cells, possibly resulting from proliferation and differentiation of progenitor cells or the conversion of principal cells into intercalated cells (Christensen et al. 2006). Zaidan et al. (2014b) suggested that similar mechanisms may be involved in the development of oncocytomas in patients on lithium therapy.

### Outcome

The possibility that tumors may be favored by long-term therapy with lithium must be weighted also in view of their outcome. It appears that the cases of thyroid cancer reported in patients treated with lithium do not differ from that found in the general population. They were invariably represented by papillary carcinoma, whose prognosis is generally considered good provided that diagnostic and therapeutic guidelines are followed (Mitchell et al. 2016). Common procedures include imaging (ultrasound and nuclear scan), fine needle aspiration biopsy, thyroidectomy, and radioiodine therapy. In our survey of the causes of death of the 375 deceased patients of the 1840 who had been visited at least once from 1980 to 2013, no cases of thyroid cancer were found. The total number of patient/years was 33,801, including 8263 patient/years on regular monitoring of lithium therapy.

With regard to renal tumors, the most common subtype in the general population is the conventional clear-cell carcinoma followed by papillary renal-cell carcinoma (Eble et al. 2004). The latter can be differentiated into two subgroups: an organ-confined/localized subgroup with a significantly better prognosis and an advanced/metastatic subgroup with a worse prognosis compared to the clear-cell carcinoma (Steffens et al. 2012).

In our survey of the causes of death, of the 375 deceased patients updated to 2013, we found only one case of renal tumor, not otherwise specified. The relationship with lithium therapy is doubtful, as this regarded a man who had taken the therapy only for a few months.

On the contrary, the case of the woman who died for metastatic oncocytoma in 2016 warrants a particular discussion. Renal oncocytomas represent 3–7% of all renal tumors. Although in the past decades malignant oncocytomas have seldom been described (Perez-Ordonez et al. 1997; Oxley et al. 2007), they are classified as benign tumors, since neoplastic cells are usually well differentiated and they are generally encapsulated and not invasive or associated with metastases (Eble et al. 2004).

Our case report is extremely unusual given the presence of lung and thyroid metastases at diagnosis. However, cases with concomitant oncocytomas and renal-cell cancers have been reported (Licht et al. 1993; Dechet et al. 1999), especially if there are diffuse nodules involving parenchyma in the same or contralateral kidney, as in the case described. We acknowledge that the histological analysis of thyroid metastasis only described the compatibility with renal origin without confirming the oncocytic nature, and no histological confirmation of the nodules in the contralateral kidney was available. Furthermore, histological diagnosis of oncocytoma may be difficult, and cases of chromophobe renal-cell cancer described as metastatic oncocytomas have been reported. Both oncocytomas and chromophobe renal-cell cancer derive from the intercalating cells of distal renal tubules, and their similar histologic features make it difficult to distinguish them, thus leading to possible misdiagnosis (Chao et al. 2002; Van der Kwast and Perez-Ordonez 2007).

### Clinical recommendations

We agree with the suggestions by Baldessarini and Tondo (2014) that tumors and other medical risks “are to be balanced against the extraordinary body of evidence that lithium, more than any other mood stabilizer, not only has short-term antimanic effects but can also reduce risks of recurrences of various components of bipolar disorder and may reduce the risks of suicide as well as of mortality associated with cardiovascular and respiratory illnesses, which are more frequent in bipolar patients compared to the general population (Bauer et al. 2006; Ahrens et al. 1995; Baldessarini et al. 2006; Grof and Müller-Oerlinghausen 2009)”. In this regard, we would like to remark our data on 375 death certificates of patients previously treated with lithium collected between 1980 and 2013 that included no cases of thyroid cancer and only one case of renal cancer (before the case identified in 2016). Even the total number of malignancies (N = 35) is outnumbered by the 64 cases of suicide, 63 of which had occurred in patients who had abandoned regular maintenance with lithium.

Nevertheless, the findings of several cases of thyroid and renal tumors as reviewed in this paper, makes us agree with the other comment on Zaidan et al results by Baldessarini and Tondo (2014): “Clinically the findings underscore the importance of close medical monitoring of patients treated with lithium, especially for more than several years” and, in particular, “consideration of renal scanning examinations for cysts and tumors among those with clinically identified, progressive impairment of renal function”.

In this regard, Rookmaaker et al. (2012) raised the question of “whether patients with chronic lithium nephropathy should be screened for renal cysts and which strategy should be applied in case a complex cyst or solid tumor is found. A subset of these tumors are oncocytomas, which are often slow-growing benign neoplasms that require only surgical intervention in the case of rapid growth or a large tumor volume (Chao et al. 2002). The benign clinical behavior challenges the indication for surgery, which results in loss of renal mass in patients with already compromised renal function. Until more information is obtained “on the incidence and natural history of lithium-related proliferative renal disease, we do not suggest radiological screening in patients with lithium nephropathy” (Rookmaaker et al. 2012).

It must be underscored that Rookmaaker et al comment dates back to 2012 before the report of excessive rates in the French nephrology department (Zaidan et al. 2014b) from one hand and the findings of rates similar to general Danish population (Kessing et al. 2015; Pottegård et al. 2016) on the other hand.

Rookmaaker et al. (2012) also suggested a biopsy “in the case of a renal mass found incidentally in a patient on chronic lithium therapy”; and “if an oncocytoma is found, careful radiological follow-up would be sufficient. If a collecting-duct-cell carcinoma is found, the same strategy could be considered in view of the benign clinical behavior of the tumours in our cases. However, treatment should be individualized and surgical removal may be preferred if a biopsy is technically not feasible, the tumour grows rapidly or removal of the tumour would imply only minimal loss of renal mass” (Rookmaaker et al. 2012).

Zaidan et al. (2014a, b) commented that “similarly to lithium-induced nephropathy, which develops slowly over decades, usually after 10–20 years, renal tumor development in lithium-treated patients may also be considered as a late complication, as assessed by a mean duration of 20 years of lithium exposure at diagnosis in our study and other reports. Thus, lithium-treated patients who have received lithium for more than 10 years and have developed CKD would be the most likely to benefit of a screening for renal tumors. Zaidan et al. (2014a, b) recommended the prescription of a renal ultrasound every 3 years. However, it is premature and unbalanced to suggest such screenings as there is simply no evidence for an increased risk of renal tumors/cancers so far.

## Conclusions

With regard to the cases of thyroid cancer, it cannot be concluded that lithium is a direct cause, because thyroid papillary carcinoma is the most common type also in the general population. No large-scale epidemiological studies have been performed. However, the prognosis is generally considered good, provided that diagnostic and therapeutic guidelines are followed.

On the other hand, the possible association between long-term lithium treatment and the occurrence of renal neoplasms is still to be assessed. So far, two population-based studies clearly showed that long-term use of lithium is not associated with increased risk of renal tumors or cancer. However, the seriousness of the alert should lead to further investigations through international cohort studies as well as implementation of the ADRs reporting.

## Abbreviations

EMA: European Medicine Agency
PRAC: Pharmacovigilance Risk Assessment Committee
ADR: adverse drug reaction
ICSRs: individual case safety reports
MedDRA: medical dictionary for regulatory activity
SOC: system organ classes
SMQs: standardized MedDRA queries
MeSH: medical subject headings
eGFR: estimated glomerular filtration rate
KDIGO: kidney disease improving global outcomes
CKD: chronic kidney disease
